# Blood Meal-Derived Heme Decreases ROS Levels in the Midgut of *Aedes aegypti* and Allows Proliferation of Intestinal Microbiota

**DOI:** 10.1371/journal.ppat.1001320

**Published:** 2011-03-17

**Authors:** Jose Henrique M. Oliveira, Renata L. S. Gonçalves, Flavio A. Lara, Felipe A. Dias, Ana Caroline P. Gandara, Rubem F. S. Menna-Barreto, Meredith C. Edwards, Francisco R. M. Laurindo, Mário A. C. Silva-Neto, Marcos H. F. Sorgine, Pedro L. Oliveira

**Affiliations:** 1 Laboratório de Bioquímica de Artrópodes Hematófagos, Instituto de Bioquímica Médica, Programa de Biologia Molecular e Biotecnologia, Universidade Federal do Rio de Janeiro, Rio de Janeiro, Brasil; 2 Instituto Nacional de Ciência e Tecnologia em Entomologia Molecular (INCT-EM), Brasil; 3 Laboratório de Bioquímica Redox, Instituto de Bioquímica Médica, Programa de Biologia Molecular e Biotecnologia, Universidade Federal do Rio de Janeiro, Rio de Janeiro, Brasil; 4 Laboratório de Microbiologia Celular, Pavilhão Hanseníase, Instituto Oswaldo Cruz, Fundação Oswaldo Cruz, Rio de Janeiro, Brasil; 5 Laboratório de Biologia Celular, Instituto Oswaldo Cruz, Fundação Oswaldo Cruz, Rio de Janeiro, Brasil; 6 Vascular Biology Laboratory, Heart Institute (InCor), University of São Paulo School of Medicine, São Paulo, Brasil; 7 Laboratório de Sinalização Celular, Instituto de Bioquímica Médica, Programa de Biologia Molecular e Biotecnologia, Universidade Federal do Rio de Janeiro, Rio de Janeiro, Brasil; Stanford University, United States of America

## Abstract

The presence of bacteria in the midgut of mosquitoes antagonizes infectious agents, such as Dengue and Plasmodium, acting as a negative factor in the vectorial competence of the mosquito. Therefore, knowledge of the molecular mechanisms involved in the control of midgut microbiota could help in the development of new tools to reduce transmission. We hypothesized that toxic reactive oxygen species (ROS) generated by epithelial cells control bacterial growth in the midgut of *Aedes aegypti*, the vector of Yellow fever and Dengue viruses. We show that ROS are continuously present in the midgut of sugar-fed (SF) mosquitoes and a blood-meal immediately decreased ROS through a mechanism involving heme-mediated activation of PKC. This event occurred in parallel with an expansion of gut bacteria. Treatment of sugar-fed mosquitoes with increased concentrations of heme led to a dose dependent decrease in ROS levels and a consequent increase in midgut endogenous bacteria. In addition, gene silencing of dual oxidase (Duox) reduced ROS levels and also increased gut flora. Using a model of bacterial oral infection in the gut, we show that the absence of ROS resulted in decreased mosquito resistance to infection, increased midgut epithelial damage, transcriptional modulation of immune-related genes and mortality. As heme is a pro-oxidant molecule released in large amounts upon hemoglobin degradation, oxidative killing of bacteria in the gut would represent a burden to the insect, thereby creating an extra oxidative challenge to the mosquito. We propose that a controlled decrease in ROS levels in the midgut of *Aedes aegypti* is an adaptation to compensate for the ingestion of heme.

## Author Summary

Mosquitoes are vectors of human pathogens, such as Dengue virus and Malaria parasites, which profoundly affect health worldwide, killing millions of people annually. Recent studies have demonstrated that the presence of bacteria in the gut of mosquitoes is able to antagonize the establishment of pathogens. Therefore, mechanisms with the potential to regulate bacterial growth in the digestive tract of mosquitoes may hamper disease transmission. Here, we show that reactive oxygen species (ROS) are present in the gut epithelia of sugar-fed mosquitoes and are drastically reduced after blood feeding through a mechanism that involves activation of protein kinase C by heme. ROS levels are inversely correlated with the presence of bacteria in the midgut and therefore we investigated if ROS are involved in fighting bacterial infections in the gut. We discovered that mosquitoes producing low levels of ROS challenged with an oral bacterial infection exhibited increased mortality due to intense bacterial proliferation and epithelial cell damage. We propose that reduction in ROS levels after blood feeding is an adaptation to compensate for the ingestion of blood, a pro-oxidant meal. This finding has consequences for the understanding of disease transmission due to the ability of ROS to modulate the gut bacterial levels.

## Introduction

Among all tissues in the insect body, gut epithelia receive the greatest exposure to microorganisms. As a consequence, complex communities of microorganisms can be found in the gut, leading to the development of a highly regulated array of immune mechanisms that mediate interactions between the insect and its microbiota [1] and influencing the transmission of pathogens by insect vectors to vertebrate hosts [2]–[4]. A major aspect of innate immunity of *Drosophila melanogaster* at the midgut interface is the production of free radicals by dual oxidases (Duox), a class of enzymes from the NOX family of proteins [5]–[8]. This is also true for mosquitoes and affects their ability to transmit human diseases such as malaria [9]–[11].

The capacity of some insect species as disease vectors is directly linked to their blood-feeding habit. An important feature of hematophagy is that huge amounts of blood are ingested by these organisms during a meal, as exemplified by *Aedes aegypti*, which in a single meal ingests volumes of blood of up to 2–3 times their pre-feeding weight. Digestion of hemoglobin, the main blood protein, inside the guts of these insects releases large quantities of its prosthetic group, heme, which has potential pro-oxidant and cytotoxic effects when not bound to proteins [12], [13]. Consequently, hematophagous arthropods need to manage the pro-oxidant effects of ingested heme after they feed on blood, as the interaction of blood-derived heme with ROS generated by the immune system would be deleterious. In fact, several protective mechanisms against heme and ROS toxicity have evolved independently in different species of blood-feeding organisms, including heme aggregation [14]–[16], heme degradation [17], [18], the expression of antioxidant enzymes [19], [20] and heme-binding proteins [21]. As a consequence, the oxidative challenge imposed by blood feeding is well circumvented by hematophagous insects, as evidenced by their extraordinary adaptive success. However, one overlooked aspect of this problem is the impact of these large amounts of heme on the redox metabolism of the midgut and, particularly on the operation of gut immune pathways connected to the production of reactive oxygen species (ROS). The concept of oxidative stress, originally defined as the imbalance between pro-oxidant compounds and antioxidant defenses, has recently been re-described as the “disruption of redox signaling and control” [22]. Related to this subject, heme notably interferes with several signaling pathways, including modulation of gene expression, protein synthesis and phosphorylation connected with cellular responses to stress [23], [24]. We have studied the effect of a blood meal on ROS levels and immune function in the midgut of *Aedes aegypti*, the vector of yellow fever and dengue virus. ROS levels in the midgut epithelia will be shown to play an important role in controlling bacteria in the midgut and is dramatically reduced soon after the ingestion of blood through a mechanism that involves PKC-dependent heme signaling.

## Materials and Methods

### Ethics statement

All animal care and experimental protocols were conducted following the guidelines of the institutional care and use committee (Committee for Evaluation of Animal Use for Research from the Federal University of Rio de Janeiro, CAUAP-UFRJ) and the NIH Guide for the Care and Use of Laboratory Animals (ISBN 0-309-05377-3). The protocols were approved by CAUAP-UFRJ under registry #IBQM001. Technicians dedicated to the animal facility at the Institute of Medical Biochemistry (UFRJ) carried out all aspects related to rabbit husbandry under strict guidelines to insure careful and consistent handling of the animals.

### Mosquitoes

*Aedes aegypti* (Red Eye strain) were raised in an insectary at the Federal University of Rio de Janeiro, Brazil, under a 12 h light/dark cycle at 28 °C and 70–80% relative humidity. Larvae were fed with dog chow, and adults were maintained in a cage and given a solution of 10% sucrose *ad libitum*. Two to ten day-old females were used in the experiments.

### Mosquito meals

Mosquitoes were artificially fed with different diets: (1) 10% sucrose (*ad libitum*), (2) heparinized rabbit blood or (3) “bicarbonate-buffered saline-agarose” (BBSA) supplemented with diverse chemicals, as indicated in the figure legends. The BBSA solution was composed of glucose (10 mg), 500 mM freshly made bicarbonate buffer pH 7.4 (10 µL), 0.5 mg low melting-point agarose and 100 mM ATP, pH 7.4 (5 µL). The final volume was set to 500 µL with 150 mM NaCl. Feeding was performed using water-jacketed artificial feeders maintained at 37 °C sealed with parafilm membranes.

### Midgut dissection and culture

Dissection was carried out in a drop of PBS at room temperature. Ten to fifteen midguts were transferred to a 24-well tissue culture flask containing 1 mL of L-15 medium supplemented with 5% fetal bovine serum without antibiotics. Midgut cultures were maintained at room temperature and were viable for at least 2 h, as assessed by the MTT reduction assay (*data not shown*) [25].

### Determination of reactive oxygen species (ROS) in the midgut

To assess ROS levels, midguts were incubated with a 2 µM solution of the oxidant-sensitive fluorophores, CM-H_2_DCFDA(5-(and-6)-chloromethyl-2′,7′-dichloro-dihydrofluorescein diacetate, acetyl ester) or dihydroethidium (hydroethidine) (DHE) *(Invitrogen)*. After a 20-min incubation at room temperature in the dark, the midguts were washed in dye-free medium, and the tissue transferred to a glass slide in a drop of PBS for epifluorescence or confocal microscopic examination. Midguts were examined with a Zeiss Axioskop 40 with an Axiocam MRC5 using a Zeiss-09 filter set (excitation BP 450–490; beam splitter FT 510; emission LP 515, for CM-H_2_DCFDA) or a Zeiss-15 filter set (excitation BP 546/12; beam splitter FT 580; emission LP 590, for DHE). Differential interference contrast (DIC) images were acquired with a Zeiss AxioObserver, which was also used for some fluorescence images, with two filter sets, Zeiss-15 and Zeiss-10 (excitation BP 450–490; beam splitter FT 510; emission BP 515–565) for CM-H_2_DCFDA. Comparison of fluorescence levels among distinct images was performed under identical conditions, using the same objectives, microscopes and similar exposure times in each experiment. Confocal images were acquired with a Zeiss LSM 510 META (Excitation at 488 nm). For hydrogen peroxide quantification, the midgut epithelia were dissected in PBS at 4 °C, the gut contents were washed out, and the tissues (pools of 10 organs) were incubated in PBS under dim light and at room temperature in the presence of 100 µM Amplex Red reagent (*Invitrogen*) and 2 units horseradish peroxidase (HRP). After 30-min incubation, the epithelia were spun, and the supernatant collected. Fluorescence (Ex: 530 nn; Em: 590 nn) was measured with a Cary Eclipse spectrofluorimeter (Varian, Palo Alto, CA, USA) and compared to a hydrogen peroxide standard curve. The total H_2_O_2_ release was corrected for non-specific oxidation of Amplex Red measured in the absence of HRP.

### HPLC analysis of superoxide in the midgut epithelium

To provide a more accurate assessment of superoxide levels, HPLC fractionation of dihydroethidium (DHE) oxidation products was used, as previously described [26]. The midgut epithelia of 20 female mosquitoes fed on sugar or 24 hours after blood meal were dissected in PBS at room temperature and incubated in L-15 medium + 5% FBS in 1.5-ml polypropylene tubes. Immediately after dissection, the midguts were spun, the supernatant removed and PBS supplemented with 150 µM dyhydroethidium (DHE) was added for 30 min at ambient temperature under dim light. In some experiments, the epithelium of sugar fed females was treated with 25 µM diphenylene iodonium (DPI) or 100 U/mL of PEG-SOD 30 min before DHE incubation. After DHE incubation, the midguts were washed twice with PBS, frozen in liquid N_2_ and homogenized. The resulting material was resuspended in acetonitrile (500 µL), sonicated (3 cycles of 8W for 5 s) and centrifuged at 2000 g for 1 min). The supernatant was dried under vacuum (SpeedVac SVC 100 – Savant), and the resulting pellet stored at −20 °C until use. Three to six pools (20 midgut epithelia/pool) were prepared, depending on the conditions. Samples were resuspended in PBS supplemented with 100 µM diethylenetriamine pentaacetic acid (DTPA) and injected into an HPLC system (Waters) equipped with a photodiode array (W2996) and fluorescence detectors (W2475). Chromatographic separation of DHE oxidation products was carried out using a NovaPak C_18_ column (3.9×150 mm, 5 µm particle size) equilibrated in solution A (water/10% acetonitrile/0.1% trifluoracetic acid) with a flow rate of 0.4 mL/min. After injection of the samples, a 0–40% linear gradient of solution B (100% acetonitrile) was applied for 10 min, followed by 10 min of 40% solution B, 5 min of 100% solution B and 10 min of 100% solution A. The amount of DHE was measured by light absorption at 245 nm, and DHE oxidation products, Hydroxyethidium (EOH) and Ethidium (E), were monitored by fluorescence detection with excitation at 510 nm and emission at 595 nm.

### RNA extraction and qPCR analysis

The protocol used was identical to Gonçalves et al. [27]. Midguts were dissected in PBS and RNA extracted using TRIzol (Invitrogen) according to the manufacturer protocol. RNA was subjected to DNAse I treatment and cDNA synthesized using High-Capacity cDNA Reverse transcription kit (Applied Biosystems). qPCR was perfomed with in a StepOnePlus Real Time PCR System (Applied Biosystems) using Power SYBR-green PCR master MIX (Applied Biosystems). The Comparative Ct Method [28] was used to compare changes in gene expression levels. A. aegypti ribosomal protein 49 gene (RP-49) was used as endogenous control. Primer sequences are given in supplementary table 1.

### dsRNA synthesis and RNAi experiments

A 964-base pair fragment from Duox gene (AAEL007563-RA) was amplified from *Aedes aegypti* with the following primers: F - GCGATCGATACATTCCGTTT and R - TTCAACAGTTCTGGCTGTCG. The amplicon was subjected to nested PCR with an additional set of primers for Duox that included T7 promoters (F - TAATACGACTCACTATAGGGATAATGTGGTCGCCAA GAGG and R - TAATACGACTCACTATAGGGTG GGACCGAACAGTTTATCC), generating a 450-base pair fragment that was used to synthesize double-stranded RNA (dsRNA) with MEGAscript RNAi kit (Ambion, Austin, TX, USA) according to the manufacturer protocol and standard mosquito RNAi settings [29]. Gene silencing experiments were performed injecting 69 nL of a 3 µg /µL solution of dsRNA into the thorax of cold-anesthetized 2 day-old female mosquitoes. Two days after injection the mosquitoes were used for experiments.

### Midgut bacterial culture and sequencing

To follow the growth profile of cultivable bacteria from the midgut of sugar or blood-fed (BF) mosquitoes, insects were surface-sterilized with 70% ethanol and dissected under aseptic conditions. Pools of 5 guts were homogenized in Luria-Bertani (LB) medium, serially diluted, plated on LB agar, allowed to grow overnight at 37 °C, and the number colony forming units (CFU) counted. One bacterial colony presenting low catalase activity (data not shown) was selected from the midgut of blood-fed mosquitoes (6 h after the meal) and grown in LB medium for subsequent experiments. Bacterial DNA was extracted with the DNeasy Blood & Tissue Kit from Qiagen according the manufacturer's instructions. DNA was subjected to PCR amplification using primers designed to amplify the 16S rDNA (Forward: 5-CCAGACTCCTACGGGAGGCAGC-3 and Reverse: 5-CTTGTGCGGGCCCCCGTCAATTC-3) (kindly provided by Dr. Carolina Barillas-Mury). The resulting product was purified, sequenced and identified using BLAST against the nucleotide collection database (nr/nt).

### Aedes aegypti feeding with bacteria and the investigation of ROS impact on the outcome of infection

Females were fed with BBSA supplemented with bacteria previously isolated from the midguts of blood-fed mosquitoes (*Enterobacter asburiae)*, as described above, in the presence or absence of 50 mM ascorbic acid (ASC) (neutralized to pH 7 with NaOH) or 10 µM DPI (diphenylene iodonium). After growing overnight in liquid LB medium, the appropriate amount of bacteria was pelleted, washed, re-suspended in 150 mM NaCl and mixed with the above components to a final volume of 500 µL. Fully engorged mosquitoes taken immediately after being fed with bacteria were transferred to new cages and scored for survival and bacterial loads at different time-points.

### Transmission electron microscopy analysis

Midguts were dissected 24 h after feeding and fixed with 2.5% glutaraldehyde in 0.1 M Na-cacodylate buffer (pH 7.2) at room temperature for 1 h before being post-fixed in 1% OsO_4_, 0.8% potassium ferricyanide and 2.5 mM CaCl_2_ in the same buffer for 1 h at 25 °C. The cells were dehydrated in an ascending acetone series and embedded in PolyBed 812 resin. Ultrathin sections were stained with uranyl acetate and lead citrate and examined in a Jeol JEM1011 transmission electron microscope (Tokyo, Japan).

## Results

### Blood feeding reduces ROS in the midgut of *Aedes aegypti*

**Figure 1:**
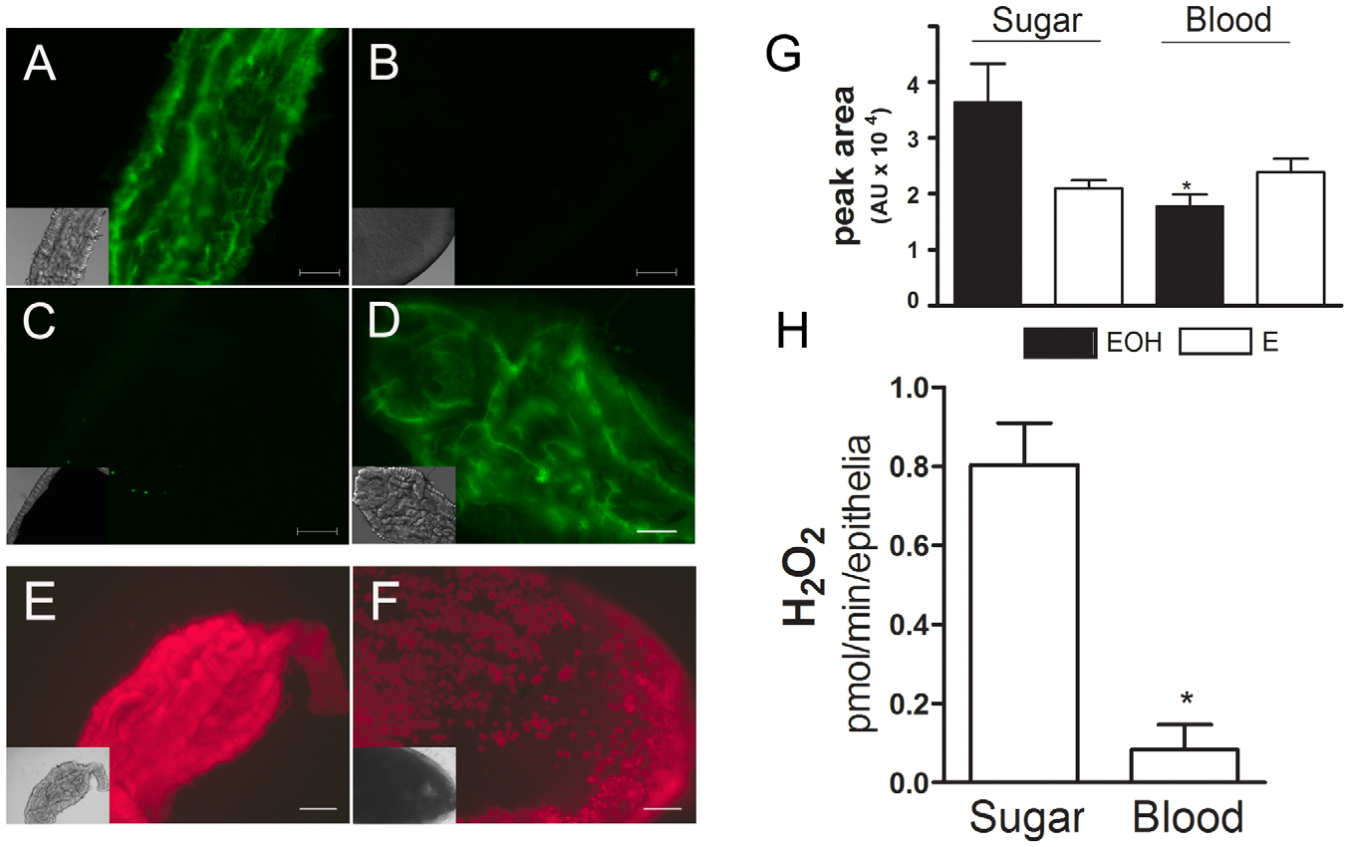
Blood meal decreases ROS levels in the midgut. Female mosquitoes were fed with sugar or blood, and midguts were dissected at different times after the meal and incubated with CM-H_2_DCFDA (2 µM) (A–D) or DHE (2 µM) (E–F) for 20 min: (A) sugar; (B) blood (0 hrs after blood ingestion); (C) blood (48 h – before excretion); (D) blood (48 h – after excretion). (E) sugar; (F) blood (24 h). The same camera exposure time was used to allow side-by-side comparison of fluorescence intensity. Differential interference contrast (DIC) images are shown as insets. Scale bar – 100 µm. (G) Superoxide radical production measured by HPLC-separation of DHE oxidation products in midgut epithelia from sugar or blood-fed mosquitoes (24 h). Asterisk indicates *P* = 0.0239 for the comparison sugar-EOH vs blood-EOH (*T*-test). EOH – 2-hydroxyethidium; E - Ethidium. (H) Hydrogen peroxide release from midgut. Asterisk indicates statistically different values (*P*<0.05, *T*-test) between sugar (n = 9) and blood-fed (n = 6) pools.

To evaluate the effect of blood meal on ROS levels in the *Aedes aegypti* midgut, four different approaches comparing sugar-fed and blood-fed females were employed. Using fluorescence microscopy and two oxidant-sensitive probes (CM-H_2_DCFDA or DHE), a robust signal was observed in the midguts of sugar-fed females (Figures 1A and 1E), an indicator of ROS. The signal was markedly reduced immediately after blood ingestion (Figure 1B), suggesting that ROS were released constitutively in sugar-fed mosquitoes and their levels decreased soon after blood intake. Oxidant levels remained low while the bolus remained in the gut (Figure 1C and 1F), but it returned to high levels immediately after excretion of feces (Figure 1D). In this panel, images were recorded immediately after excretion, giving fluorescence signals comparable to those in sugar-fed females.

Although redox-sensitive dyes have been extensively used in fluorescence microscopy to study biological roles of ROS, their ability to identify the nature of the oxidizing species has been questioned due to the lack of selectivity of most probes *in vivo* [30], [31], including DCF-based probes [32]. With DHE, its oxidation by different reactive species results in the formation of distinct products that can be separated by HPLC. Two main compounds are formed: ethidium (E), which is most likely formed by reaction with more than one oxidant species, and 2-hydroxyethidium (EOH), shown to be a reliable indicator of the presence of superoxide [32]–[34]. The amount of ethidium (E) was relatively constant throughout all conditions tested (Figure 1G), while the levels of 2-hydroxyethidium were higher in sugar-fed mosquitoes, corroborating the results obtained by microscopy and demonstrating that superoxide is one of the major reactive species found in gut epithelia from sugar-fed insects. This experiment was performed with midgut epithelia (free of gut content), supporting the conclusion that ROS are generated by epithelial cells. In addition, EOH signal was specifically reduced in sugar-fed midguts both by incubation with DPI, an inhibitor of the flavin-containing NADPH oxidase, and PEG-SOD (supplementary figure S1). We also investigated alternative sources of ROS/RNS through the incubation of midguts from sugar-fed mosquitoes with inhibitors of nitric oxide synthase and xanthine oxidase, respectively L-NAME and allopurinol, but none of these reagents were able to decrease ROS signal in the midgut (supplementary figure S2).

It is well known that superoxide spontaneously or enzymatically, through the action of SOD, forms hydrogen peroxide (H_2_O_2_)_,_ a diffusible ROS. We measured H_2_O_2_ release in the midgut and detected higher levels in the epithelium of sugar-fed mosquitoes when compared to blood-fed insects (Figure 1H). Altogether, the data in Figure 1 strongly support the conclusion that at least superoxide and hydrogen peroxide are present in the midgut of *Aedes aegypti* females fed on sugar and that a blood meal results in reduced ROS levels.

### ROS is released by midgut epithelial cells at the luminal surface in sugar-fed mosquitoes

**Figure 2:**
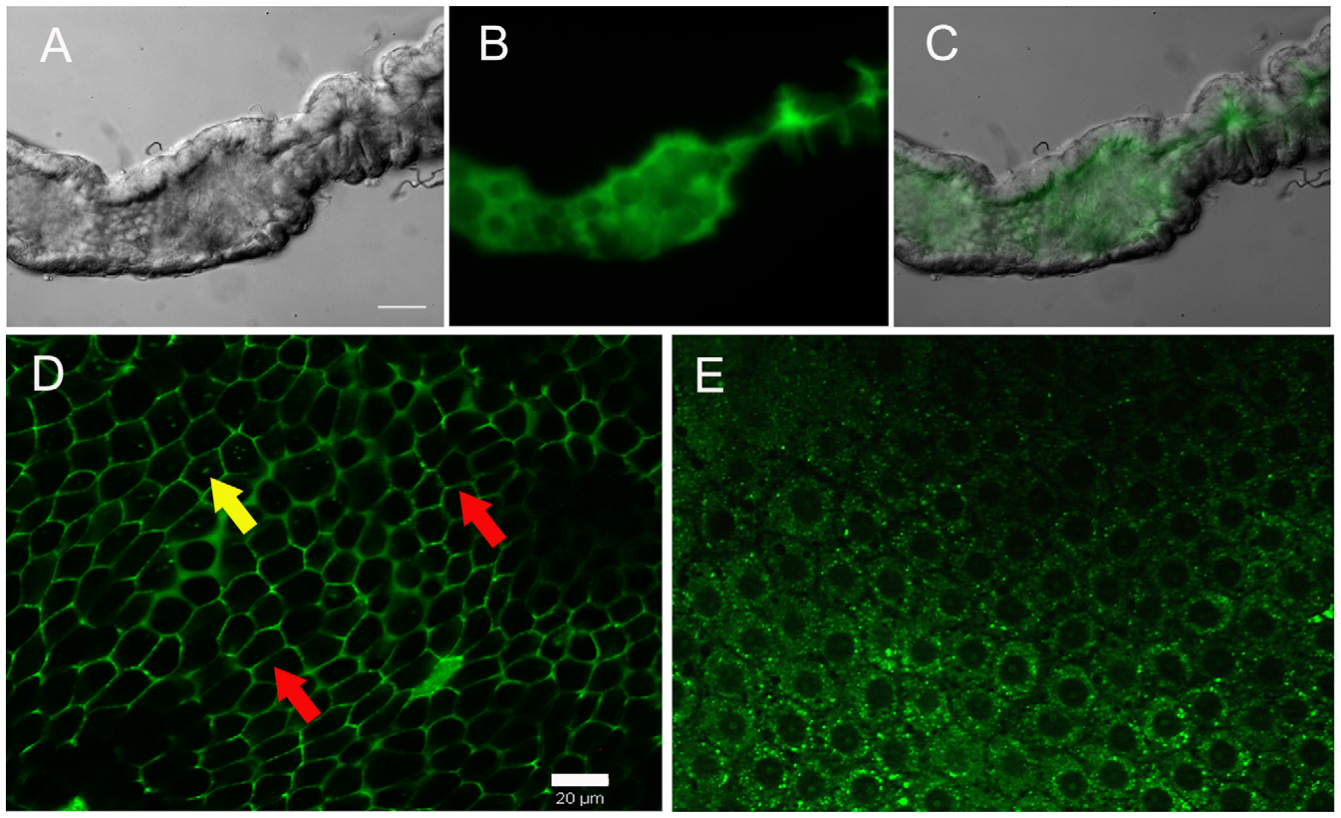
Sites of ROS location in the midgut epithelia. Midguts were incubated with CM-H_2_DCFDA (2 µM) for 20 min, washed and visualized under an epifluorescence (A–C) or confocal microscope (D–E). (A) DIC image of sugar-fed midgut. Scale bar– 100 µm. (B) ROS staining of midgut in (A). (C) Merge of (A) and (B). (D) Confocal image of epithelia from sugar-fed or (E) blood-fed (24 hrs) females showing different patterns of ROS production. Scale bar – 20 µm. The fluorescence intensity is not directly comparable in the images shown in panels D and E because the microscope was set to acquire the best image under each condition to allow optimal visualization of cellular ROS production sites. Red arrows indicate fluorescence associated with the cell periphery. Yellow arrows indicate fluorescence associated with intracellular organelles.

CM-H_2_DCFDA fluorescence was not uniformly distributed throughout the gut, being strongly concentrated in the lumen (Figure 2A–C), further evidenced by confocal microscopy, which showed an intense ROS signal at the lumenal surface in a longitudinal optical section of the midgut (supplementary figure S3). Thus, most of the ROS was generated by epithelial cells and released into the lumen. Transverse optical slices taken at the apical region of the midgut epithelium of sugar-fed mosquitoes showed a honeycomb-like appearance, indicating that ROS were located mostly at the periphery of epithelial cells (Figure 2D – red arrows). After a blood meal, overall CM-H_2_DCFDA oxidation was strongly reduced (Figure 1), cells were flattened due to midgut distension, and fluorescence associated with intracellular organelles was observed (Figure 2E – yellow arrows). It is important to emphasize that the overall fluorescence intensity in Figure 2D was much greater than that in 2E, as shown in the previous results in which sugar-fed and blood-fed CM-H_2_DCFDA fluorescence had been compared (Figure 1). Panels 2D and 2E have a similar intensity because the microscope setup was adjusted to acquire the best image under each condition to allow for the best localization of ROS. A careful examination of Figure 2D shows that intracellular fluorescence, probably associated with organelles, is also seen in the epithelia of sugar-fed mosquitoes (Figure 2D – yellow arrow).

### Decrease in ROS signal after a blood meal is triggered by both hemoglobin and heme, which is mediated by PKC

**Figure 3:**
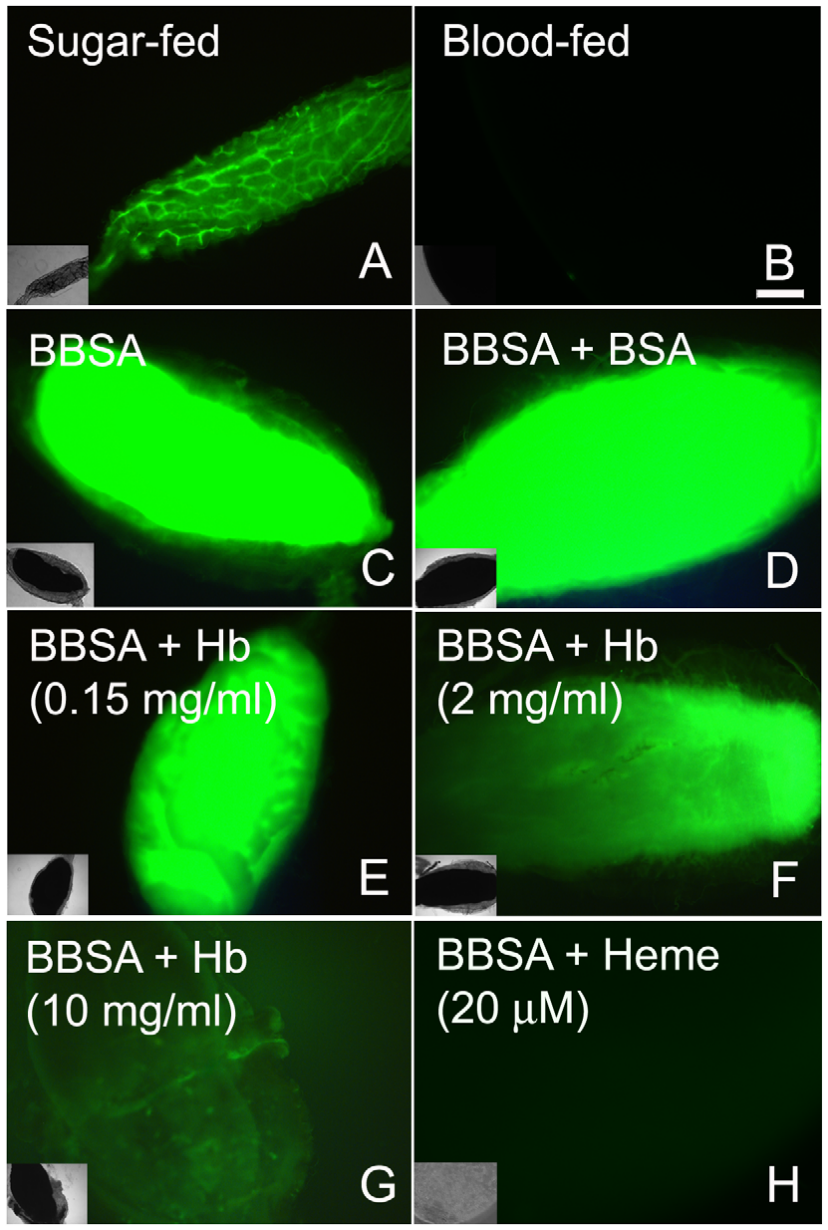
Heme is responsible for blood-induced ROS inhibition in the midgut. Female mosquitoes were fed different meals: (A) sugar; (B) blood (dissected immediately after meal); (C) BBSA alone; (D) BBSA + albumin (BSA) (50 mg/mL); (E–G) BBSA + hemoglobin; (H) BBSA + heme (20 µM). Midguts shown in panels B-H were dissected immediately after feeding. CM-H_2_DCFDA fluorescence is shown in all panels. Insets are differential interference contrast (DIC) images. Scale bar – 100 µm.

**Figure 4:**
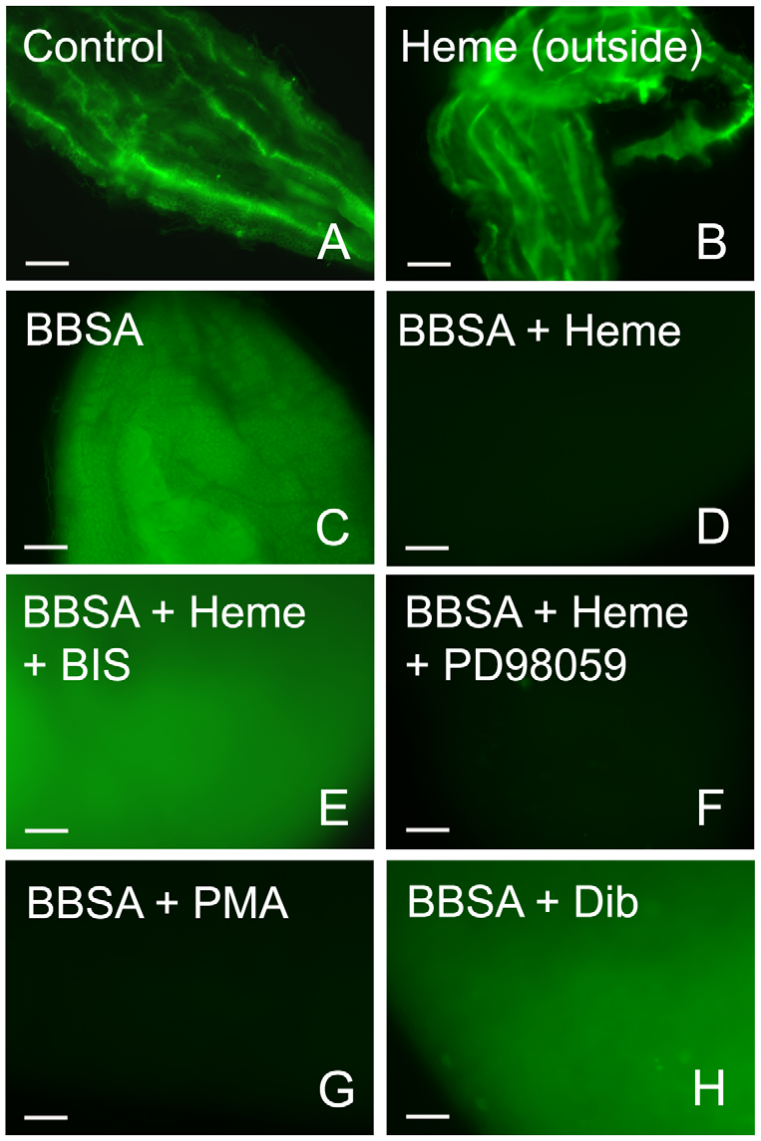
Heme-induced inhibition of ROS is mediated by PKC. ROS signal from the midgut under different conditions was evaluated. (A) Sugar-fed mosquitoes (B) Sugar-fed midguts incubated with heme (20 µM) added in the culture medium. In Figures C–H, female mosquitoes were fed with BBSA supplemented with different chemicals as indicated and dissected immediately after feeding. (C) BBSA alone; (D) BBSA + heme (20 µM); (E) BBSA + heme (20 µM) + BIS (10 nM) (PKC inhibitor). (F) BBSA + heme (20 µM) + PD98059 (50 µM) (MAPK inihibitor). (G) BBSA + PMA (100 ng/ ml) (PKC agonist). (H) BBSA + dibutyryl-cAMP (50 µM) (PKA agonist). These fluorescent images were acquired using the same microscope setup to allow direct comparison of signal intensities. Representative images are shown.

An incoming blood meal decreases ROS levels, as shown above in Figure 1 and 3A–B. Feeding insects on a solution containing salts and low melting-point agarose, referred hereafter as “BBSA” (see Materials and Methods) resulted in CM-H_2_DCFDA fluorescence that was comparable (Figure 3C) or even higher than sugar-fed midguts (Figure 3A), suggesting that midgut distention is not responsible for decreasing ROS levels after feeding. Interestingly, the addition of hemoglobin to BBSA decreased ROS in a dose-dependent manner, with almost complete suppression of oxidation of the probe at 10 mg/mL, equivalent to 7% of the total blood hemoglobin concentration after a full meal (Figures 3E–G). Feeding mosquitoes with BBSA enriched with albumin, the main plasma protein, did not reduce ROS levels, showing that this effect was specific to hemoglobin (Figure 3D). Most important, a low concentration of heme alone added to the BBSA solution reduced ROS to levels similar to those observed after a complete blood meal (Figure 3H and 4H). Further confirmation that the fluorescence under this experimental condition was due to the presence of ROS came from suppression of fluorescence signals after feeding females with BBSA supplemented with the antioxidants N-acetyl-cysteine (NAC) or uric acid (supplementary figure S4).

To gain insight into the mechanism that mediates heme-induced decrease of ROS, mosquitoes fed with BBSA supplemented with heme (Figure 3H and 4D) showed lowered ROS levels similar to that observed after a regular blood meal (Figure 3B). Interestingly, sugar-fed midguts incubated with heme added in the culture medium showed no reduction of fluorescence signal (Figure 4B), with their ROS levels remaining the same as in sugar-fed midguts alone (Figure 4A). As heme has previously shown to activate PKC both in vertebrates and invertebrate models (42, 43), as well as modulate MAP kinase activity (44), we have explored a possible involvement of PKC in the pathway that decreases ROS levels after a blood meal. Feeding insects with heme together with bisindolylmaleimide (BIS), an inhibitor of protein kinase C (PKC) [35], prevented inhibition of ROS by heme (BIS in Figure 4E). However, decrease of ROS by heme was not reversed by feeding with PD98059 (Figure 4F), a MAPK inhibitor [36], demonstrating the specific involvement of PKC in this pathway. Furthermore, PKC activation through PMA supplementation of BBSA lowered ROS levels, even in the absence of heme (Figure 4G). In contrast, feeding with dibutyryl-cAMP (Dib in Figure 4H), an agonist of PKA (42), did not change ROS levels, demonstrating the specific involvement of PKC activation, but not MAPK and PKA, in heme-mediated down-regulation of ROS. DIC images from the midguts used in Figure 4 are also shown (supplementary figure S5).

### Reduced ROS levels in the midgut allows proliferation of intestinal microbiota

**Figure 5:**
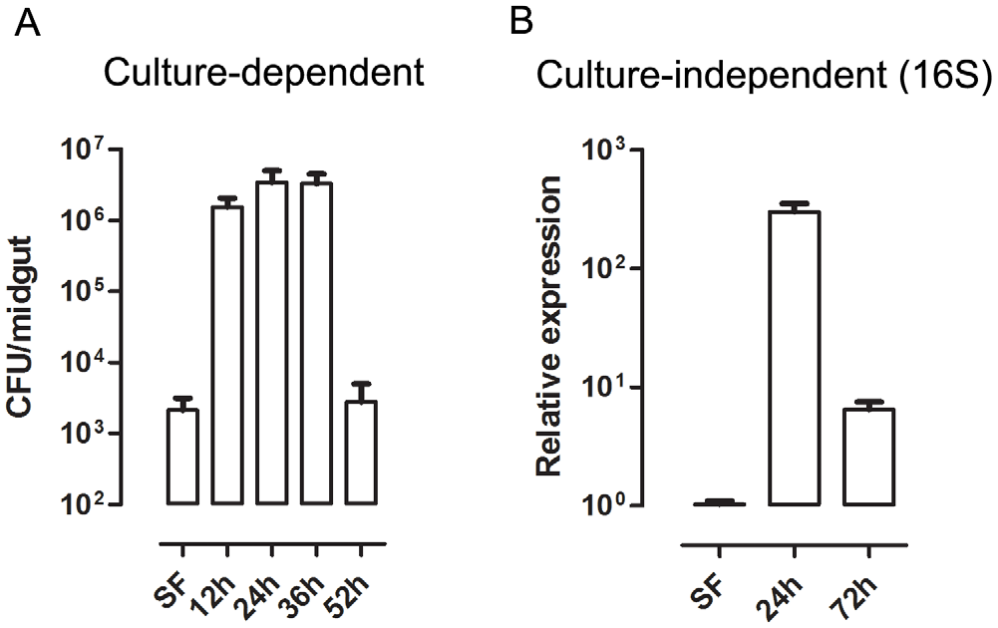
Time-course of microbial growth in *Aedes aegypti* midgut before and after a blood meal. (A) Evaluation of cultivable bacterial population in sugar-fed (SF) and blood-fed midguts dissected at different times after feeding. (B) Culture-independent evaluation of microbiota in sugar-fed (SF) and blood-fed midguts through qPCR for bacterial ribosomal 16S RNA [54].

**Figure 6:**
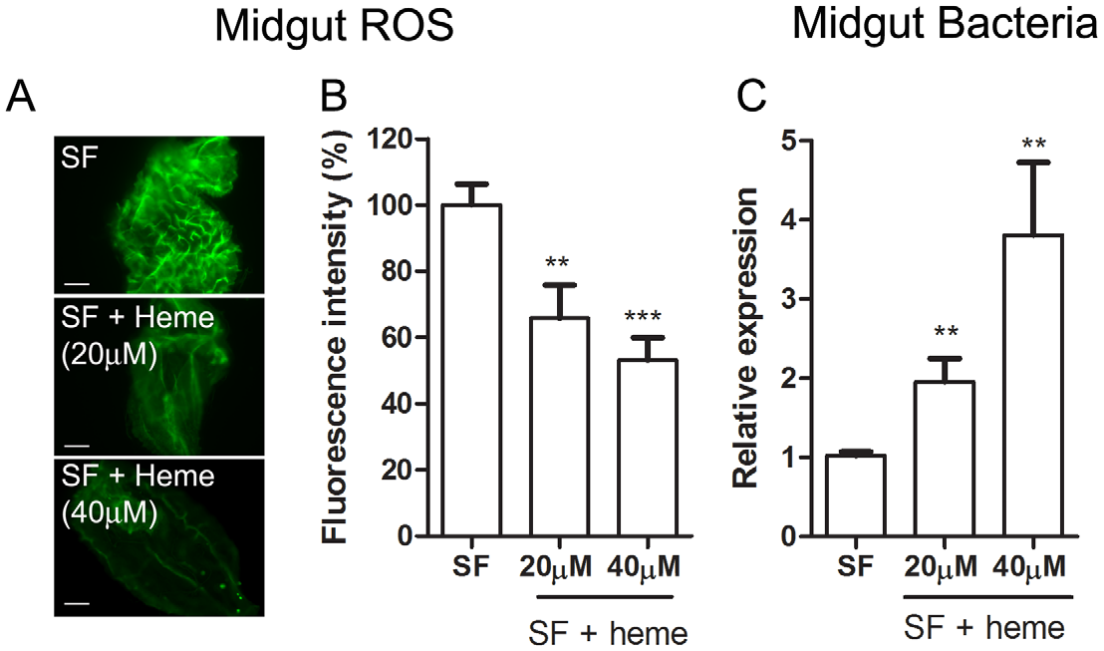
Heme decreases ROS levels in the midgut and allow proliferation of intestinal microbiota. Female *Aedes aegypti* were fed *ad libitum* for five days with sucrose supplemented with heme (20 and 40 µM). (A) Representative images of midgut ROS after heme treatment. Scale bar – 100 µm. (B) Fluorescence associated with individual midguts was quantified with ImageJ software. The number of midguts used was: SF – 24, SF + heme (20 µM) – 23, SF + heme (40 µM) – 21. ** *P* < 0.01. *** *P* < 0.0001 (ANOVA followed by Tukey's Multiple Comparison Test – GraphPad Prism). (C) Gut bacteria was assessed through qPCR from pools of 10 midguts. 8 pools from each condition were used. ** *P*<0.01 (T-test – GraphPad Prism).

**Figure 7:**
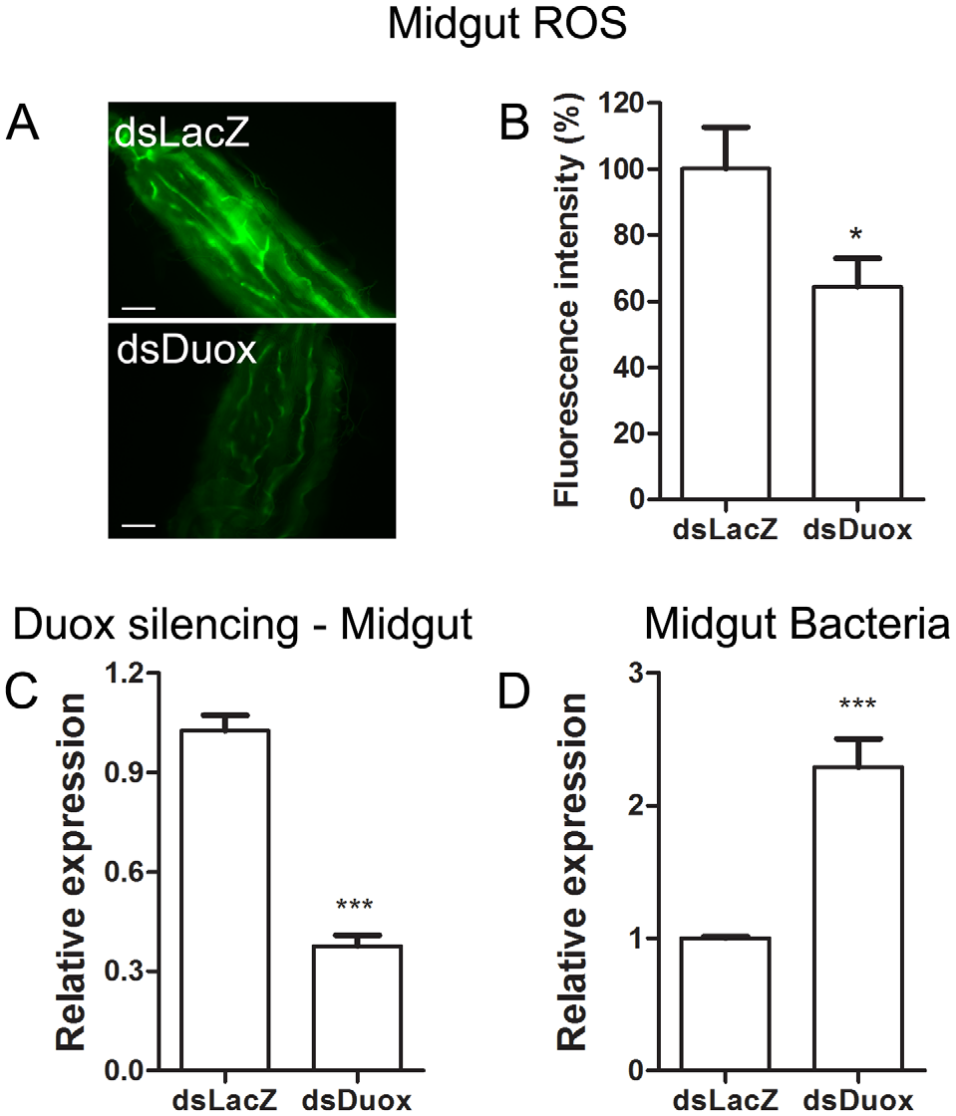
Duox silencing in the midgut reduces ROS levels and allow proliferation of intestinal microbiota. 2-day old female *Aedes aegypti* were injected with dsDuox or a non-related control dsRNA (dsLacZ). 2 days later individual mosquitoes were subjected to evaluation of midgut ROS (A–B) or pools of 10 mosquitoes were used for midgut RNA extraction followed by qPCR analysis (C–D). (A) Representative images of midgut ROS after dsDuox injection. Scale bar – 100 µm. (B) Fluorescence associated with individual midguts was quantified with ImageJ software. The number of midguts used was: dsLacZ – 13, dsDuox – 26. * *P*<0.05 (T-test – GraphPad Prism). (C) qPCR analysis of Duox expression in the midgut revels a silencing efficiency of 67% compared to LacZ-treated groups. *** *P*<0.0001 (T-test – GraphPad Prism). (D) Gut bacteria was assessed through qPCR from pools of 10 midguts. 8 pools from each condition were used. *** *P*<0.001 (T-test – GraphPad Prism).

The gut bacteria population was investigated using both CFU counting (cultivable bacteria) and a culture-independent method (16S RNA quantization by real-time PCR). These methods produced similar profiles, with an increase of ∼3 orders of magnitude (Figure 5) at 24 h after a blood meal (ABM), with an inverse correlation being found between the proliferation of bacteria in the gut and ROS levels. Female mosquitoes fed *ad libitum* for 5 days with heme (diluted in sucrose solution) demonstrated a dose-dependent decrease in ROS levels in the midgut (Figure 6A–B) and a concomitant increase in intestinal microbiota (Figure 6C). This result provides a demonstration that heme decreased ROS levels in the midgut which created a favorable environment for bacterial growth. Ha *et al.* [6] showed that the production of ROS by a Duox enzyme has an important role in controlling bacterial proliferation in the gut of *Drosophila*. RNAi-mediated gene silencing of Duox in the midgut of *Aedes aegypti* (Figure 7C) also resulted in reduced ROS levels (Figure 7A–B) and increased intestinal microbiota (Figure 7D), revealing that Duox is a major source of ROS in sugar-fed *Aedes aegypti.* Consistently, most of ROS signal is located at the periphery of gut epithelial cells, which is expected for Duox enzymes (Figure 2D – red arrow), and is distinct from localization of ROS in blood-fed epithelia, which is basically associated to intracellular organelles (Figure 2E – yellow arrows). Mosquitoes treated with antibiotics still exhibited an intense ROS signal associated to the midgut, despite having their gut flora reduced by >90% (data not shown).

### Redox modulation of pathogenesis after bacterial infection in *Aedes aegypti*

**Figure 8:**
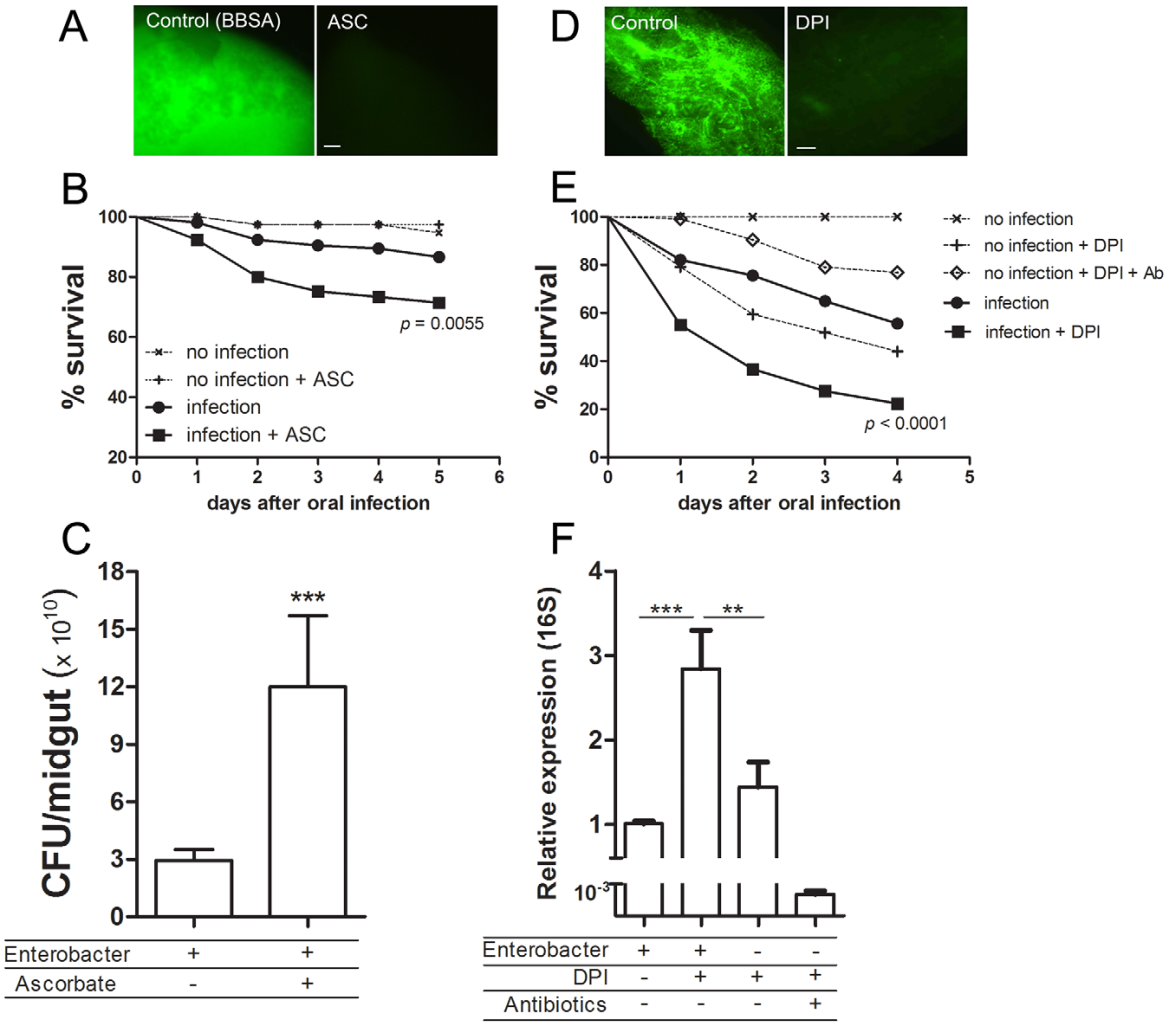
Redox modulation of bacterial growth in the midgut. (A) Female mosquitoes were fed with BBSA + ascorbic acid (ASC) (50 mM pH 7) and immediately dissected and ROS levels were determined based on CM-H_2_DCFDA fluorescence. (B) Females were fed with BBSA + gut commensal bacterium *Enterobacter asburiae* at a concentration of 2×10^9^ CFU/mL with or without ascorbic acid (50 mM) and were scored for survival daily. Data was analyzed with the Log-rank Test (GraphPad Prism 5). *P* = 0.0055 between “infection” and “infection + ASC”. The result shown is the sum of 3 independent experiments. Total number of mosquitoes: Control (n = 40). Control + ASC (n = 40). Infection (n = 105). Infection + ASC (n = 105). (C) In a different group of experiments using the same infection setup as in Figure 7B, mosquitoes were surface sterilized and dissected (5 midguts/pool; 24 h after bacterial oral infection) and the number of CFU/midgut was determined. Figure 8C is the result of a representative experiment using 15 pools per condition. *** *P*<0.0001 following a Kolmogorov-Smirnov Comparison test. (D) Sugar-fed midguts were pre-incubated for 1 h in medium alone (control) or medium supplemented with diphenylene iodonium (DPI, 10 µM) and stained for ROS with CM-H_2_DCFDA. Scale represents 100 µm. (E) Females were fed with BBSA supplemented *Enterobacter asburiae* at a concentration of 4×10^9^ CFU/mL with or without DPI (10 µM), as well as BBSA + DPI with or without antibiotics (penicillin/streptomycin/tetracycline – 200 U/mL, 200 µg/mL, 100 µg/mL, respectively) and scored for survival daily. The total number of mosquitoes was 123 for “infection” 120 for “infection + DPI”, 30 for “no infection”, 106 for “no infection + DPI” and 120 for “no infection + DPI + Ab”. The result shown is the sum of 3 independent experiments. *P*<0.0001 for the comparison between “infection” and “infection + DPI (Log-rank Test – GraphPad Prism). (F) RNA from whole body (minus head) of pools of 5 mosquitoes was extracted and quantification of bacterial 16S RNA was performed using 6 independent pools of mosquitoes. ** *P*<0.01. *** *P*<0.0001 (ANOVA followed by Tukey's Multiple Comparison Test – GraphPad Prism).

We decided to investigate whether ROS modulates the ability of *Aedes aegypti* to fight a bacterial oral infection. Females were fed a sub-lethal dose of a bacteria, identified as *Enterobacter asburiae* based on 16S rDNA sequencing (supplementary figure S6), isolated from the midgut of blood-fed (6 h ABM) insects from our colony. Figure 8B shows that mosquitoes orally infected with this bacteria in the presence of ascorbic acid (BBSA + Enterobacter + ASC), a condition that reduces ROS levels (Figure 8A), had a significantly decreased life span compared to the group infected in the absence of the antioxidant. Concomitantly, there was a 4-fold increase in the amount of bacteria in the midgut 24 h after the bacteria-containing meal (Figure 8C), suggesting that increased mortality might be attributable to increased proliferation of bacteria in the midgut. *In vitro* growth of *Enterobacter asburiae* in the presence of ascorbic acid did not result in increased proliferation of bacteria after 24 h of culture in LB media (data not shown), demonstrating that the increased bacterial growth in mosquitoes infected in the presence of ascorbate (Figure 8C) was due to the absence of ROS. This conclusion was supported by feeding mosquitoes with *Enterobacter asburiae* together with DPI (BBSA + Enterobacter + DPI), which also inhibited ROS production (Figure 8D and supplementary figure S1), causing a marked increase in mortality (Figure 8E), accompanied by a 3-fold increment in the proliferation of bacteria 24 h after challenge when compared to mosquitoes that ingested *Enterobacter asburiae* only (without DPI) (Figure 8F). Noteworthy, mosquitoes fed *ad libitum* with DPI (diluted in sucrose) without *Enterobacter asburiae* exhibited no mortality (data not shown) but showed increased proliferation of endogenous gut flora (supplementary figure S7) when compared to sugar-fed mosquitoes. In Figure 8E–F it is also shown that feeding mosquitoes with BBSA + DPI (without Enterobacter) caused significant mortality (“no infection + DPI” in Figure 8E) and increased bacterial counting (Figure 8F). Endogenous bacterial growth and mortality was reverted by antibiotic treatment demonstrating that mortality was due to bacterial proliferation (“no infection + DPI + Ab” in Figure 8E).

**Figure 9:**
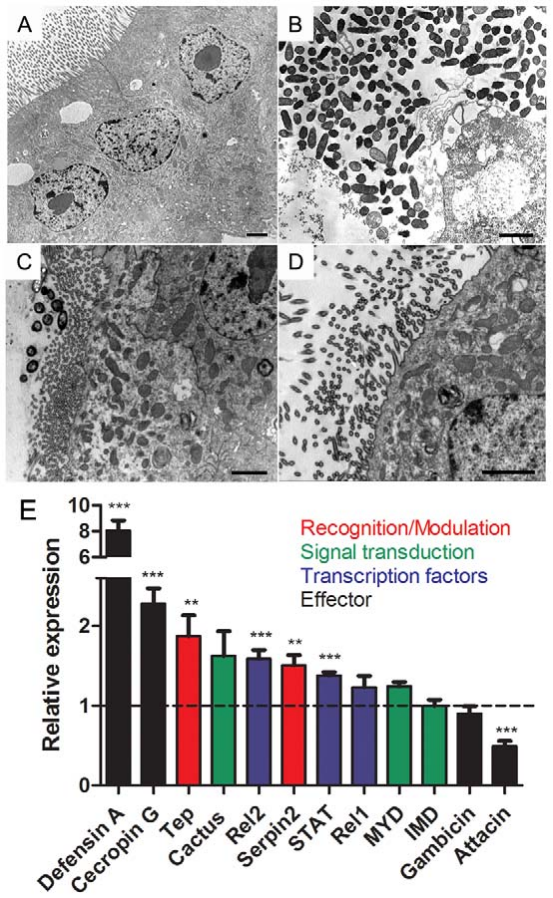
Bacterial infection in the presence of DPI causes cell damage and immune activation in mosquito midgut. Mosquitoes were fed with BBSA with *Enterobacter asburiae* at a concentration of 4×10^9^ CFU/mL with or without DPI (10 µM). 24 h later midguts were dissected and processed for transmission electron microscopy. (A) Mosquitoes infected with bacteria only. (B) Mosquitoes infected with bacteria + DPI (10 µM) to reduce ROS. (C) Mosquitoes fed with BBSA + DPI. (D) Mosquitoes fed with BBSA + DPI + antibiotics (penicillin/streptomycin/tetracycline – 200 U/mL, 200 µg/mL, 100 µg/mL, respectively). All the scale bars represent 2 µm. (E) Gene expression analysis of whole body (minus head) mosquitoes 24 h after feeding with BBSA + bacteria + DPI. Dashed line indicates gene expression of mosquitoes fed with BBSA + bacteria (without DPI). Different classes of immune genes are indicated with colors.

We used transmission electron microscopy (TEM) in mosquito midguts to explore in more detail the pathogenic mechanisms responsible for increased mortality 24 hours after bacterial infection in the presence of DPI. Mosquitoes infected with *Enterobacter asburiae* without DPI had healthy epithelial cells, including a normal aspect of the microvilli and cytoplasm (Figure 9A). In contrast, midguts from mosquitoes fed with bacteria and DPI presented highly damaged epithelia, with loss of microvilli and aberrant cell morphology (Figure 9B). In figure 9C it is shown the integrity of cells and the presence of some bacteria, that is morphologically distinct from *Enterobacter asburiae* (supplementary figure S8), in the lumen after feeding mosquitoes with DPI alone (BBSA + DPI), suggesting the proliferation of bacteria from the gut flora due to the lack of ROS after DPI treatment. In comparison, the group treated with DPI and antibiotics (BBSA + DPI + Ab) displayed a healthy midgut (Figure 9D). The immune status of mosquitoes fed with bacteria with or without DPI was evaluated through gene expression analysis and revealed a complex response to infection. Figures 9E and supplementary figure S9 demonstrates that some anti-microbial genes were up-regulated (cecropin and defensin) while attacin was down-regulated after DPI treatment. This result highlights the complexity of mosquito innate immune response, involving the concerted action of multiple effectors such as ROS and antimicrobial peptides.

## Discussion

Overall the data shows that ROS are continuously present in the midgut of sugar-fed *Aedes aegypti* female mosquitoes and that a blood meal immediately decreased ROS through a mechanism that involves heme-mediated PKC activation. This event occurred in parallel to the expansion of gut bacterial levels, which led the hypothesis that ROS is involved in the control midgut bacteria. The presence of heme or silencing of Duox resulted in decreased ROS levels and increased proliferation of endogenous bacteria. Finally, using a model of bacterial infection in the gut, we showed that the absence of ROS resulted in decreased mosquito resistance to infection and increased mortality. Gut epithelial cells constitute the surface of the body of all metazoans most exposed to contact with microorganisms. These microorganisms comprise a variety of species and ecological relationships, from symbiosis to pathogenicity, and an array of immunological mechanisms essential for the control of the intestinal microbial population have been described. The production of reactive species is one of the key players in gut immunity. Ha et al. [5], [6] showed that the redox balance in the gastrointestinal tract of *Drosophila melanogaster* is a major microbial control system, determining whether a fly lives or dies after oral infection with bacteria. Two components have been identified, a Duox enzyme that generates ROS to oxidize and kill microbes, and an immune-regulated extracellular catalase that removes any excess of luminal ROS that might harm the gut epithelia of the fly. In the malaria vector, the mosquito *Anopheles gambiae*, it was recently described that after a blood meal the concerted action of Duox and a peroxidase is required to form a dityrosine barrier that decreases midgut permeability to bacterial elicitors, preventing immune activation and creating a favorable environment for plasmodium development [11]. In addition, control of levels of hydrogen peroxide seems to directly modulate the immune responses against both bacteria and *Plasmodium* [10]. Similar ROS-mediated immune responses have been described in *Caenorhabditis elegans* [37] and *Manduca sexta* [38].

Here, we provide for the first time direct evidence that in *Aedes aegypti* superoxide anion – and hence hydrogen peroxide – is produced by epithelial cells and secreted into the lumen of the midgut (Figures 1 and 2). ROS levels were inversely correlated with the occurrence of bacteria in the midgut (Figure 1, 5, 6 and 7), and the presence of ROS increased mosquito survival after an oral challenge with bacteria (Figure 8). However, a unique feature of the mosquito midgut is that a dramatic decrease in ROS levels occurs after a blood meal (Figures 1). If sugar-fed mosquitoes adopt the same pattern of intestinal immunity as in other insects, it is not clear why they should behave differently after a blood meal, renouncing the use of ROS as a major weapon to regulate the growth of gut bacteria. The explanation probably resides in the fact that the pro-oxidant activity of heme released in the gut upon digestion of hemoglobin interacts and converts lipid hydroperoxides (ROOH), which exhibit quite low reactivity, into the highly reactive peroxyl (ROO^−•^) and alcoxyl (RO^−•^) radicals that have very pronounced cytotoxicity [39]–[41]. Lipid hydroperoxides are normally produced due to abstraction of electrons from lipids by reactive species produced by metabolic pathways, such as respiration in mitochondria, or as a consequence of immune-related oxidase action. Therefore, heme alone does not generate ROS; it only converts pre-formed oxidized molecules back into highly reactive intermediates in the lipid peroxidation chain, thus acting as a catalyst for the formation of potentially toxic radicals. Thus, we propose that after blood feeding, *Aedes aegypti* shuts down ROS generation to avoid heme-mediated oxidative stress. Consequently, ROS-based immunity is greatly reduced after a blood meal, contributing to bacterial proliferation.

Recognizing this phenomenon as an important adaptation that attenuates heme toxicity led us to investigate the signaling mechanism triggering the down-regulation of ROS after a blood meal. Midgut distention can be excluded as a potential mechanism because fully engorged insects fed with BBSA showed intense CM-H_2_DCFDA fluorescence (Figure 3C). Although hemoglobin is able to decrease ROS, heme alone can account for down-regulation of ROS levels (Figure 3, panel H). The fact that this effect is observed upon early exposure to the incoming diet (<20 min) excluded mechanisms based on modification of gene expression and led us to search for the involvement of protein kinases, a hypothesis that was confirmed by preventing the heme-mediated suppression of ROS with a PKC inhibitor (Figure 4E) and by mimicking the effect of heme using a PKC activator (Figure 4G). In this regard, heme-induced reduction in ROS levels was only found when heme was located in the apical (Figure 4D) but not the basal side of the midgut epithelial cells (Figure 4B), revealing that this signaling pathway was triggered specifically through a mechanism that activated PKC after sensing heme in the lumen, which was achieved by feeding, but not by incubating heme in the culture medium. Alternatively, we cannot exclude the possibility that the result obtained in Figure 4B may reveal that the gut does not respond to heme *in vitro*. It was already shown that the synthesis of uric acid by *Rhodnius prolixus* fat body could be triggered by heme through activation of PKC [42], suggesting conservation of this signaling pathway. Activation of PKC by heme modulates ROS production in human neutrophils [43], [44]. Curiously, in these cells heme was a positive effector of ROS production, suggesting that, although the heme capacity to activate PKC is probably conserved in this signaling pathway, a modification downstream of this protein kinase leads to suppression of ROS in the mosquito midgut instead of activation. This hypothesis is a major target for future research.

Gut bacteria experience an explosion in growth after ingestion of a blood meal by a mosquito (Figure 5). A simple explanation would be that the proliferation of bacteria after blood feeding is favored by the increase in availability of nutrients compared to sugar-fed mosquitoes (data not shown). Our data suggest that bacterial proliferation is also stimulated by the down-regulation of ROS levels. In spite of the fact that the reduction in ROS levels was sufficient to increase the gut flora, none of these treatments was able to allow the growth of endogenous bacteria to levels found after a blood-meal (100–1000 times more bacteria), probably due to the lack of nutrient supply to support microbial growth. This conclusion is further supported by the data in Figure 6 and 7, where reduced ROS levels due to the presence of heme or RNAi-mediated silencing of Duox resulted in proliferation of endogenous bacteria. ROS reduction occurring in the presence of ascorbate or DPI, 2 unrelated antioxidants that decrease ROS levels through different modes of action (Figure 8), also resulted in increased bacterial proliferation, leading to tissue damage and increased mortality in insects given a sub-lethal dose of a bacterial species naturally found in the gut. A large amount of work has been done on invertebrate immunity, especially in *Drosophila melanogaster* and mosquitoes, since the discovery that Toll and IMD pathways play a paramount role in the defense against invading microorganisms [45], [46]. However, knockout of the IMD pathway alone in *Drosophila* leads only to modest alterations in survival when infected orally with ROS-susceptible bacteria [5], but this NF-κB pathway was essential in insects challenged with ROS-resistant microbes [47]. In this regard, it is interesting that the bacteria we used, an *Enterobacter* (Gram-negative) isolated from the midgut, whose growth was favored by reduced ROS levels (Figure 8), had low levels of catalase (data not shown) similar to most species of this genus [48], and was also found in the gut of *Anopheles gambiae* (3) In a similar way to that described for *Drosophila* [47], the immune response triggered in our infection system was not entirely based on production of ROS, but included the up-regulation of the antimicrobial peptides, cecropin and defensin (Figure 9E), known to be part of the IMD pathway [49], and are responsive to Gram-negative (G-) bacterial infection [50]. However, not all genes related to immune response behave in the same way and several genes did not show significant activation. Reduced expression of attacin, which is involved in the defense against gram-negative bacteria [51], [52], prompts us to speculate that attacin down-regulation may be part of the pathophysiological mechanism(s) involved in increased mosquito mortality. When ROS production was blocked by DPI, there was bacterial proliferation in the gut and several antimicrobial genes were up-regulated (Figure 9E and S9), in a possible attempt to reduce tissue damage induced by the bacteria. Immune genes are overexpressed after a blood meal (data not shown) and this could compensate for reduction of ROS levels reported here, explaining why mosquitoes do not die after blood intake, in spite of having increased bacteria proliferation together with the lack of a major antibacterial mechanism. Taken together, these results highlight a complex effect of the blood meal on the immune regulation network.

This work has several consequences for the biology of insects that are vectors of disease. One is that a similar phenomenon may operate in the guts of other blood-feeding insects, a possibility currently being studied in our laboratory. The other is that it has the potential to influence infection rates of pathogens transmitted by insect vectors. In fact, a strain of *Anopheles gambiae* that is refractory to *Plasmodium* infection lives in a chronic state of oxidative stress [9]. At first glance, one might expect that the decrease in midgut ROS levels after blood meal would be beneficial for the establishment of viral or protozoan infections. However, this situation allows bacterial growth a condition that antagonizes Dengue and *Plasmodium* infections [2], [3].

Our results are also in line with the hypothesis we proposed a few years ago, namely, that while degrading hemoglobin, some hematophagous organisms such as the blood fluke, *Schistosoma mansoni*, and *Plasmodium* parasites decrease ROS generation by shifting energy metabolism to a glycolysis-based anaerobic mode in order to avoid heme-induced oxidative stress [53]. Interestingly, this effect seems to not only affect the midgut but also may constitute a systemic trend, because respiration and H_2_O_2_ generation in *Aedes* flight muscle mitochondria are also reduced following a blood meal [27].

Our data provide a novel view of ROS production in the midgut of a disease vector, highlighting the complexity of the mosquito immune response, where the decrease in ROS generation that comes with hematophagy creates a favorable environment for bacterial proliferation, with possible implications for a better understanding of molecular mechanisms that influence vector competence.

## Supporting Information

Figure S1 Modulation of superoxide radical by DPI and PEG-SOD. Sugar-fed midguts were pre-incubated in the presence of either PEG-SOD (100 U/mL) (Sigma) or DPI (25 µM) for 30 min and transferred to medium with DHE for 20 min; the DHE oxidation products were measured by HPLC. * P<0.0001 for the comparison between sugar and sugar + PEG-SOD or Sugar + DPI (ANOVA, followed by Dunnetts multiple comparison test).
(0.49 MB TIF)

Figure S2 Nitric oxide synthase and xanthine oxidase inhibitors do not decrease ROS in sugar-fed midguts. (A) L-NAME (1 mg/mL) or (B) allopurinol (500 µM) was added to sugar-fed midgut cultures for 1 h at room temperature, and ROS levels were evaluated under the microscope using CM-H_2_DCFDA.
(1.27 MB TIF)

Figure S3 ROS produced by midgut epithelial cells is released into the lumen in sugar-fed mosquitoes. (A) ROS staining with CM-H_2_DCFDA in the midgut of sugar-fed mosquitoes. The image shows a longitudinal optical section of the midgut. Scale bar- 50 µm. Black asterisk indicates an air bubble in the gut lumen. (B) The same experimental setup as in “A” showing the gut at a lower magnification. Blue represents DAPI (nuclear stain). Scale bar - 20 µm
(1.18 MB TIF)

Figure S4 ROS modulation by different antioxidants. Female mosquitoes were fed with BBSA alone (A) or BBSA supplemented with 20 mM N-acetyl-cysteine (NAC) (B) (solubilized in 200 mM Tris-buffer) or 500 µM urate (C) and immediately dissected. ROS levels were determined based on CM-H_2_DCFDA fluorescence. Scale bar represents 100 µm.
(2.03 MB TIF)

Figure S5 Differential interference contrast images of midguts from experiment shown in Figure 4.
(1.46 MB TIF)

Figure S6 16S DNA gene sequence from *Enterobacter asburiae* isolated from the midgut of *Aedes aegypti*. Females had their midguts dissected 6 h after a blood meal before being plated on LB agar. One colony with low catalase activity was isolated; PCR of the 16S gene was performed after DNA extraction and the sequencing data are shown. BLAST analysis of the 1026-bp fragment allowed identification of the bacterial colony as *Enterobacter asburiae* (accession number AJ506159), a gram-negative bacteria known to be weakly reactive to the catalase test [48].
(2.06 MB TIF)

Figure S7 Mosquitoes were fed *ad libitum* with sucrose 5% supplemented 10 µM DPI for 5 days and RNA was extracted from the midgut and processed for 16S quantification through qPCR.
(0.27 MB TIF)

Figure S8 Transmission electron microscopy from the bacterial population typically found in the gut of *Aedes aegypti* 24 hours after feeding mosquitoes with BBSA + *Enterobacter asburiae* + DPI (left) or BBSA + DPI (right).
(0.67 MB TIF)

Figure S9 Mosquitoes were fed with BBSA + DPI (10 µM) or BBSA + DPI + antibiotics (penicillin/streptomycin/tetracycline). 24 h later RNA from whole body (minus head) was extracted and gene expression was performed by qPCR. Dashed line indicates gene expression of mosquitoes fed with BBSA + bacteria (without DPI), similar to Figure 9E. Different classes of immune genes are indicated with colors. * *P*<0.05, ** *P*<0.01. *** *P*<0.0001 after t-test comparing each condition with mosquitoes fed with BBSA + bacteria (without DPI).
(1.31 MB TIF)
