# Correction: Blood Meal-Derived Heme Decreases ROS Levels in the Midgut of *Aedes aegypti* and Allows Proliferation of Intestinal Microbiota

**DOI:** 10.1371/annotation/34fed6f4-4096-4980-bf2f-b918e14f5ff5

**Published:** 2013-02-26

**Authors:** Jose Henrique M. Oliveira, Renata L. S. Gonçalves, Flavio A. Lara, Felipe A. Dias, Ana Caroline P. Gandara, Rubem F. S. Menna-Barreto, Meredith C. Edwards, Francisco R. M. Laurindo, Mário A. C. Silva-Neto, Marcos H. F. Sorgine, Pedro L. Oliveira

Table S1 does not appear. Please view Table S1 here: 
