# Supplementary figures and images for: Blood Meal-Derived Heme Decreases ROS Levels in the Midgut of Aedes aegypti and Allows Proliferation of Intestinal Microbiota
Source: PLoS Pathog. 2011 Mar 17;7(3):e1001320. doi: 10.1371/journal.ppat.1001320 (PMC3060171; doi:10.1371/journal.ppat.1001320)

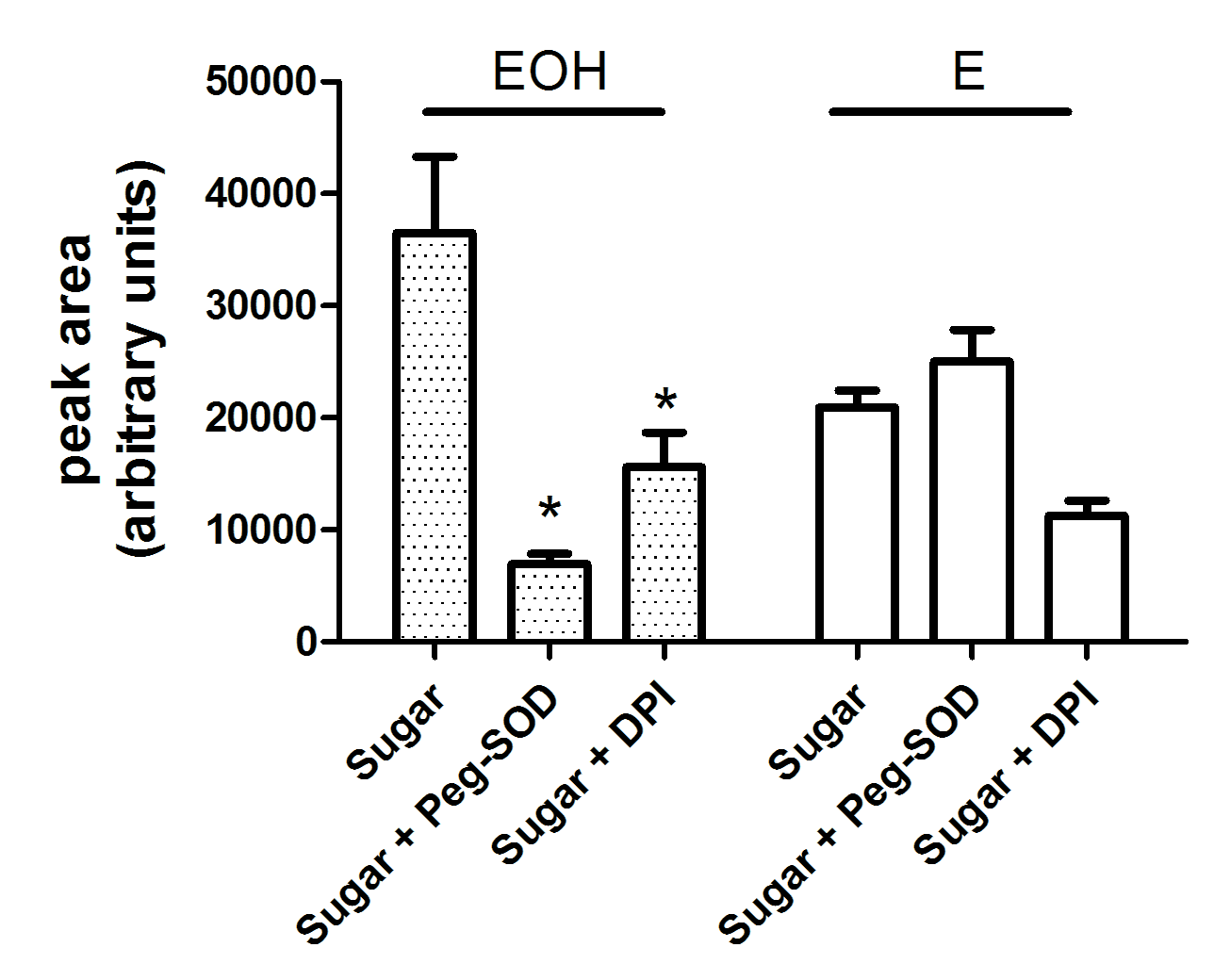

Supplement: Figure S1 — Modulation of superoxide radical by DPI and PEG-SOD. Sugar-fed midguts were pre-incubated in the presence of either PEG-SOD (100 U/mL) (Sigma) or DPI (25 µM) for 30 min and transferred to medium with DHE for 20 min; the DHE oxidation products were measured by HPLC. * P<0.0001 for the comparison between sugar and sugar + PEG-SOD or Sugar + DPI (ANOVA, followed by Dunnetts multiple comparison test). (0.49 MB TIF) [file ppat.1001320.s001.tif]

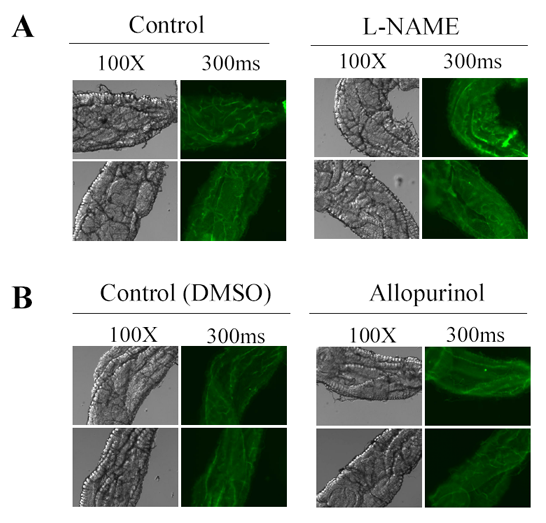

Supplement: Figure S2 — Nitric oxide synthase and xanthine oxidase inhibitors do not decrease ROS in sugar-fed midguts. (A) L-NAME (1 mg/mL) or (B) allopurinol (500 µM) was added to sugar-fed midgut cultures for 1 h at room temperature, and ROS levels were evaluated under the microscope using CM-H2DCFDA. (1.27 MB TIF) [file ppat.1001320.s002.tif]

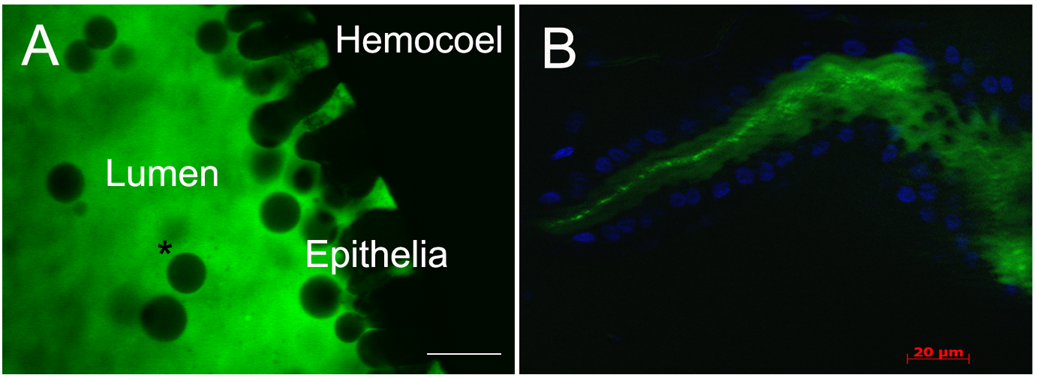

Supplement: Figure S3 — ROS produced by midgut epithelial cells is released into the lumen in sugar-fed mosquitoes. (A) ROS staining with CM-H2DCFDA in the midgut of sugar-fed mosquitoes. The image shows a longitudinal optical section of the midgut. Scale bar- 50 µm. Black asterisk indicates an air bubble in the gut lumen. (B) The same experimental setup as in “A” showing the gut at a lower magnification. Blue represents DAPI (nuclear stain). Scale bar - 20 µm (1.18 MB TIF) [file ppat.1001320.s003.tif]

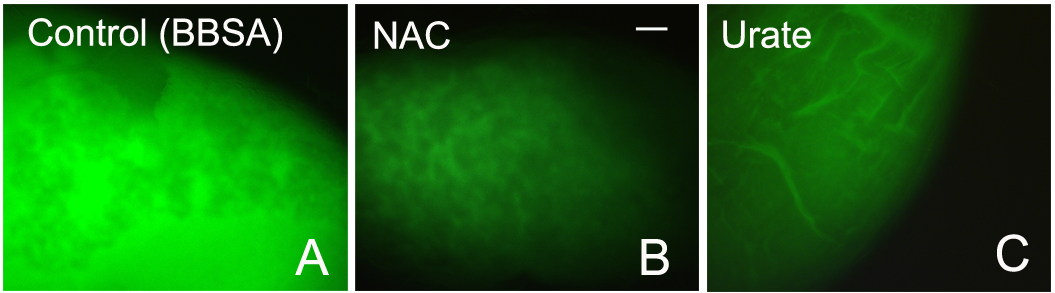

Supplement: Figure S4 — ROS modulation by different antioxidants. Female mosquitoes were fed with BBSA alone (A) or BBSA supplemented with 20 mM N-acetyl-cysteine (NAC) (B) (solubilized in 200 mM Tris-buffer) or 500 µM urate (C) and immediately dissected. ROS levels were determined based on CM-H2DCFDA fluorescence. Scale bar represents 100 µm. (2.03 MB TIF) [file ppat.1001320.s004.tif]

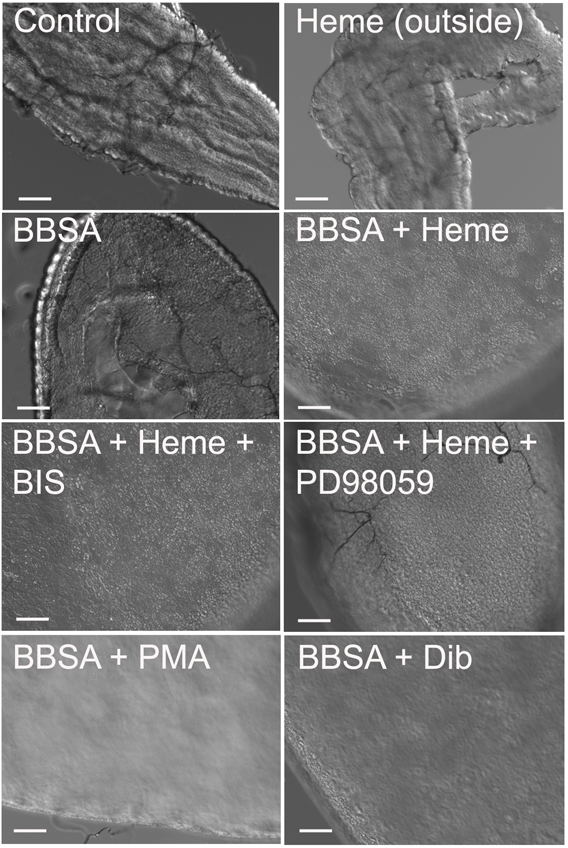

Supplement: Figure S5 — Differential interference contrast images of midguts from experiment shown in Figure 4. (1.46 MB TIF) [file ppat.1001320.s005.tif]

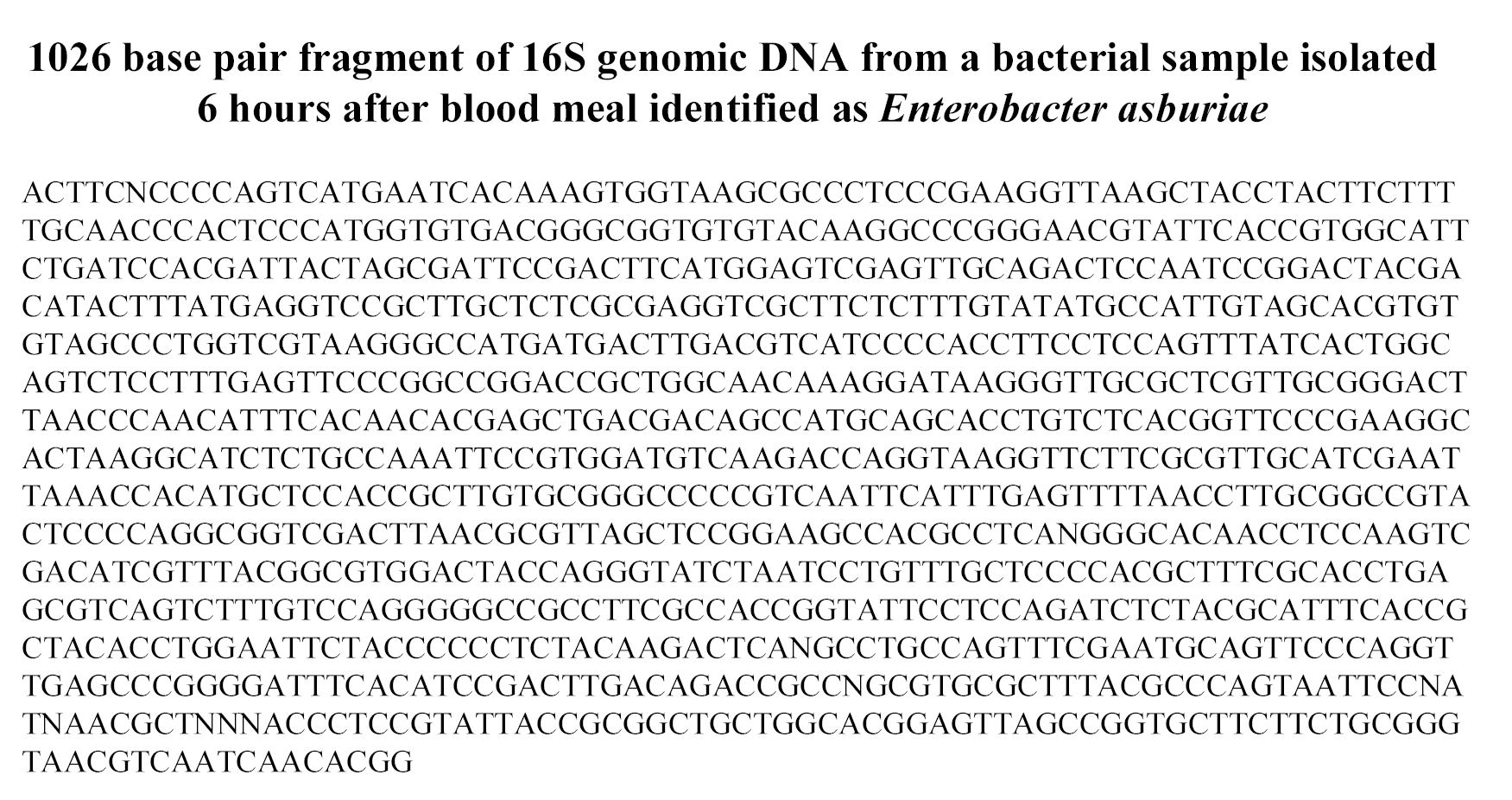

Supplement: Figure S6 — 16S DNA gene sequence from Enterobacter asburiae isolated from the midgut of Aedes aegypti. Females had their midguts dissected 6 h after a blood meal before being plated on LB agar. One colony with low catalase activity was isolated; PCR of the 16S gene was performed after DNA extraction and the sequencing data are shown. BLAST analysis of the 1026-bp fragment allowed identification of the bacterial colony as Enterobacter asburiae (accession number AJ506159), a gram-negative bacteria known to be weakly reactive to the catalase test [48]. (2.06 MB TIF) [file ppat.1001320.s006.tif]

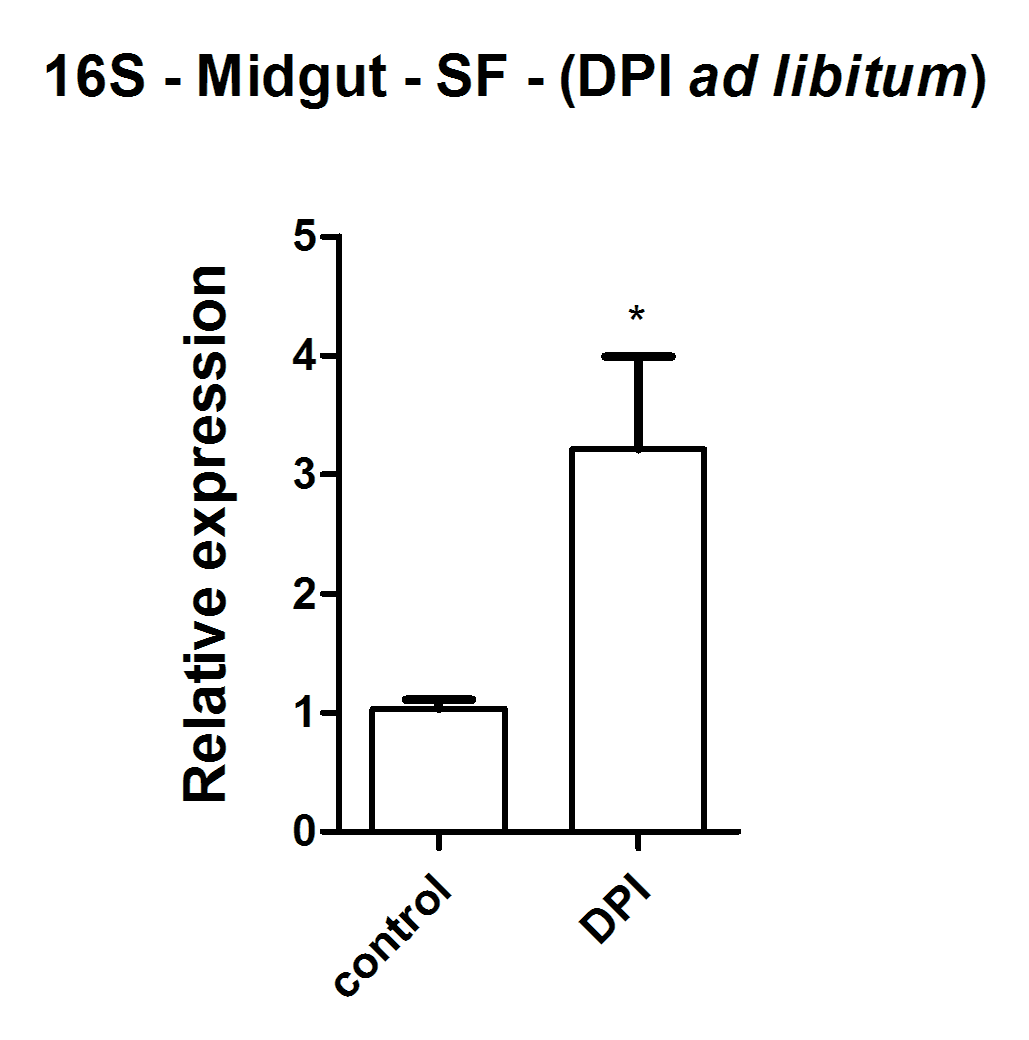

Supplement: Figure S7 — Mosquitoes were fed ad libitum with sucrose 5% supplemented 10 µM DPI for 5 days and RNA was extracted from the midgut and processed for 16S quantification through qPCR. (0.27 MB TIF) [file ppat.1001320.s007.tif]

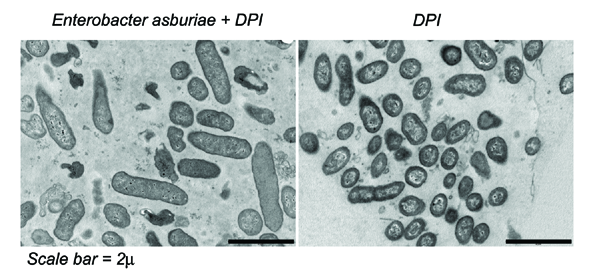

Supplement: Figure S8 — Transmission electron microscopy from the bacterial population typically found in the gut of Aedes aegypti 24 hours after feeding mosquitoes with BBSA + Enterobacter asburiae + DPI (left) or BBSA + DPI (right). (0.67 MB TIF) [file ppat.1001320.s008.tif]

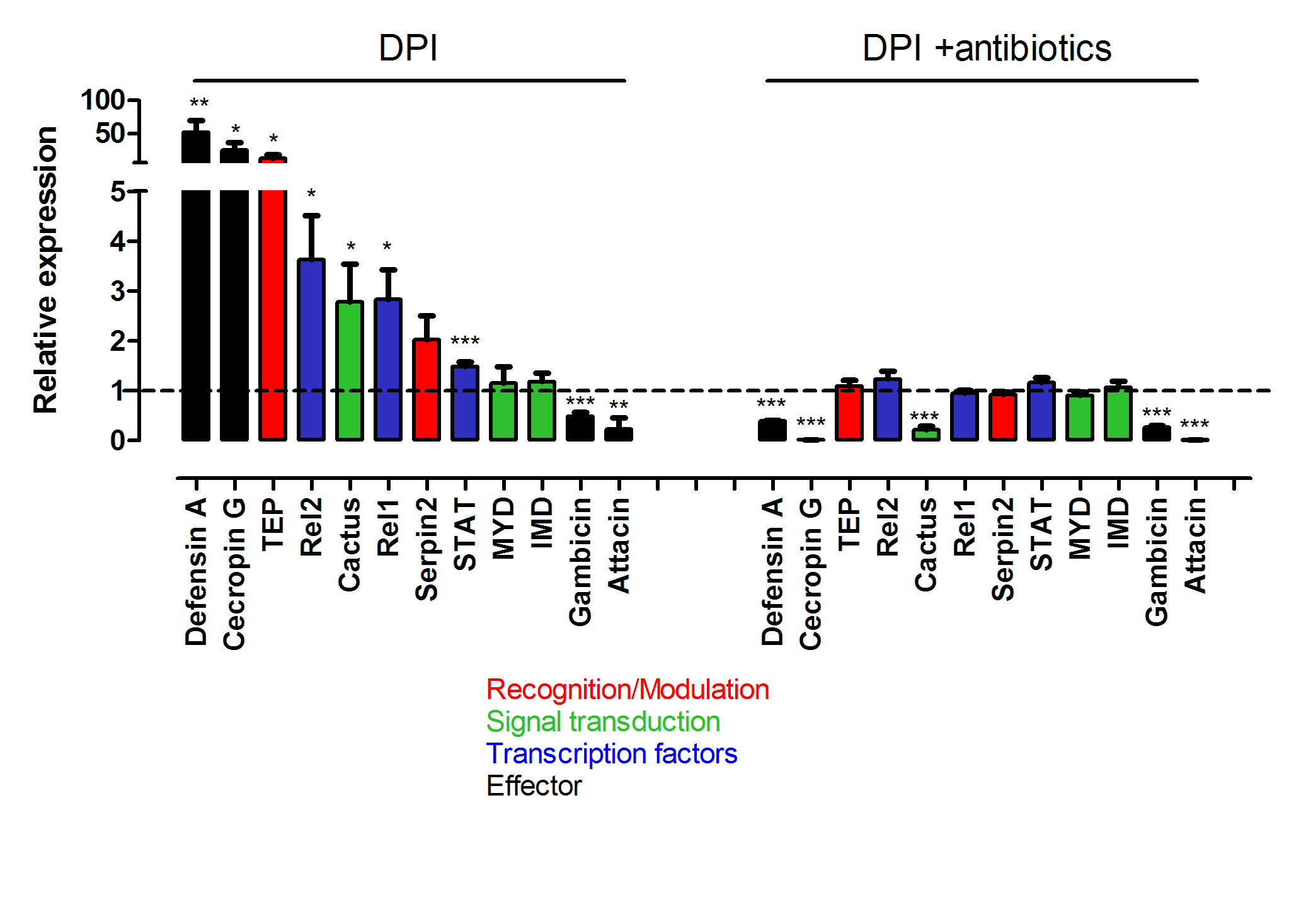

Supplement: Figure S9 — Mosquitoes were fed with BBSA + DPI (10 µM) or BBSA + DPI + antibiotics (penicillin/streptomycin/tetracycline). 24 h later RNA from whole body (minus head) was extracted and gene expression was performed by qPCR. Dashed line indicates gene expression of mosquitoes fed with BBSA + bacteria (without DPI), similar to Figure 9E. Different classes of immune genes are indicated with colors. * P<0.05, ** P<0.01. *** P<0.0001 after t-test comparing each condition with mosquitoes fed with BBSA + bacteria (without DPI). (1.31 MB TIF) [file ppat.1001320.s009.tif]
